# Exploring the Impact of the Somatic Method ‘Timani’ on Performance Quality, Performance-Related Pain and Injury, and Self-Efficacy in Music Students in Norway: An Intervention Study

**DOI:** 10.3389/fpsyg.2022.834012

**Published:** 2022-02-23

**Authors:** Anna Détári, Tina Margareta Nilssen

**Affiliations:** ^1^Department of Music, University of York, York, United Kingdom; ^2^Musicians’ Health and Movement Institute, Oslo, Norway

**Keywords:** musician’s health, intervention, somatic method, young musicians, performance quality, performance-related pain and injury

## Abstract

The importance of including performance-related body mechanics into music education to improve performance quality and prevent performance-related injuries has been stressed by many researchers recently. However, it is unclear how this information could be delivered most effectively. The somatic method ‘Timani’ provides a practical solution by combining expertise in music performance and functional body mechanics with the goal of achieving a more efficient playing technique. Since no in-depth study has been conducted to assess the method before, we explored the impact of this method on young musicians’ performance through an online, 4-week-long Timani intervention with a mixed-methodology design. 17 students (mean age = 19.17 years) were recruited from the Norwegian Academy of Music. They participated in two group workshops at the beginning and the end of the project and received four individual Timani sessions administered by certified teachers. We collected survey data at the workshops about performance-related pain and self-efficacy, and qualitative feedback after each session. In addition, all sessions were observed by the researcher and semi-structured interviews were conducted with the teachers about the perceived outcomes and their experiences with teaching the method online. Our findings show that the intervention had a positive impact on a physical, professional, and to some extent, psychological and behavioral level. The improvements included better posture when playing, enhanced control and dexterity in the upper extremities, and improved breathing mechanisms. The seven students who had performance-related pain pre-intervention reported a reduction in the discomfort. The positive results were achieved by the dual expertise of the teachers in music performance and functional body mechanics, the structure of the sessions, the communication, and the pedagogical tools used. Both students and teachers felt that administering the sessions online was satisfactory and produced good results. Timani is a promising method to establish healthy playing and singing habits thus improving performance quality and preventing performance-related problems and has great potential in reducing pre-existing injuries and pain. Also, it can be effectively taught online which has further implications for the logistics of delivery.

## Introduction

Playing a musical instrument or singing is often romanticized as a purely artistic pursuit, overlooking the fact that it is as much of physical activity as well as a creative and aesthetic one. Musicians have to perform extremely complex motor movements when playing or singing, which requires very refined patterns of muscle activation (Furuya and Altenmüller, 2015). They are often called the athletes of the small muscles (Quarrier, 1993), yet their knowledge and education about body mechanics and their preparation for the physical side of the task is less than satisfactory, and rarely comparable to those pursuing sports on a high level (Chan and Ackermann, 2014).

While there are initiatives to include biomechanical analysis in musical performance (Kjelland, 2000; Visentin et al., 2008; Kelleher et al., 2013), the majority of musicians are relying on their primary instrumental or singing teacher when learning the complex coordination and posture needed for music-making. This educational process, however, places much more attention on the auditory output than the physical movements the musician uses to achieve it (Chan and Ackermann, 2014). Moreover, the information shared on lessons about the performance-related body mechanics is rarely based on an up-to-date anatomical understanding or founded on biomechanical principles; teachers are likely to replicate their own education (Mills and Smith, 2003) or base their tuition on their own personal experience as musicians and educators (Visentin et al., 2008), which subsequently, might result in less-than-ideal body mechanics and instrumental technique in some students.

This missing piece in professional music tuition can contribute to various performance-related problems (Visentin et al., 2008). One subtle and often overlooked outcome is that inefficient body mechanics and posture can negatively affect the sound production, lead to limitations in the technical skill, and thus, inhibit musical expression and hinder musicians to reach their full potential (Ueno et al., 1998; Baadjou et al., 2017; Shoebridge et al., 2017).

Yet, working on playing-related body mechanics and posture is rarely used as a tool to enhance performance quality, the topic comes to the forefront of the conversation much more often when it leads to a performance-related injury that thwarts the instrumental or singing skill (Steinmetz et al., 2010). This is not surprising, given the high prevalence of such injuries in this population in all age groups from college-level music students (Cruder et al., 2020) to adult professionals (Kok et al., 2016).

However, young musicians might downplay the consequences of such injuries (Stanhope, 2018) and the possibility of honing their skills might motivate them more to engage with intervention programs rather than the idea of injury prevention. Musicians generally tend to take a task-specific view and are more motivated to do additional physical exercises when they perceive it as directly related to their instrumental technique (Ackermann et al., 2002) and are interested in practical, and instrument-specific physical and somatic education (Stanhope, 2018). Shoebridge et al. (2017) also highlight that the right posture, which allows efficient coordination with minimal effort, needs to be achieved in a dynamic performance context.

Yet, the majority of the interventions and preventative programs presented in the literature are usually underpinned by the narrative of avoiding getting injured or aiming for better overall health and do not include task-specific exercises. The focus is often on improving general strength and endurance through exercise and fitness programs, sometimes even focusing on one specific body part, such as the trunk or the upper extremities (Stanhope et al., 2020). Apart from the fact that the evidence is not consistent in terms of the safety and effectiveness of these interventions and they can possibly worsen the condition of the musician (Stanhope et al., 2020), and despite the reported positive outcomes in terms of flexibility, ease of the movements, and muscle strength, some of these interventions were unable to show a significant positive effect on the endurance and perceived exertion in performance and practice situations (Chan et al., 2014a,b).

In addition to exercise programs, several somatic methods have been tested on musicians with the goal of relieving performance-related problems and enhancing the body mechanics and instrumental technique when playing. These methods are developed with the general population in mind and tailored to the specific needs of musicians. Examples of this in the literature include Alexander Technique (AT) (Kleinman and Buckoke, 2013; Klein et al., 2014), Feldenkrais Method (FM) (Lee, 2018), Body Mapping (Buchanan and Hays, 2014) and yoga and yogic breathing (Khalsa and Cope, 2006; Lee et al., 2016).

These studies have various outcome measures and methodologies with varying quality. Some are only descriptive and do not include any experimental data (Lee, 2018), with the aim to provide some guidelines for teachers to incorporate the methods into music lessons. What they all have in common, however, is that they are generally taking a more holistic approach and stress the importance of improving general posture and alignment over targeting specific muscle groups or general strength and endurance (Schlinger, 2006).

Although these methods are gaining more and more popularity among musicians, there is not much data available on how they translate these methods’ ideas to dynamic performance situations. From the 12 controlled trial AT studies Klein et al. (2014) reviewed, most used AT sessions away from the instrument as an intervention and allowed the participants to incorporate the learned material into their playing on their own. Similarly, providing general group ATM (awareness through movement) sessions is the core component of most FM interventions for musicians (Beacon et al., 2017; Paparo, 2021) with few direct links introduced between the FM methods and playing mechanics.

The general difficulty is that many of these methods have been developed for commonly held postures and performed movements, such as sitting or walking, while musicians need to use additional instrument-specific body mechanics for sound production. When interacting with the instrument, general alignment and movement patterns change; Doyle (1984) found that after a short AT intervention, 98.6% of both the experimental and control group of violinists still changed their head and neck posture when taking the instrument up, following their habitual movement patterns.

Extrapolating from this, to make an intervention more effective, it seems necessary to correct and practice the body mechanics and posture in a dynamic performance context. This approach is currently lacking from the literature, with most of the interventions working with exercises away from the instrument and only little available data on how to transfer the embodied experience of alignment and effective body mechanics to a dynamic playing situation.

The somatic method ‘Timani’ seems to meet many of these needs and challenges. It was created by pianist, massage therapist, yoga teacher, personal trainer, and Kinetic Control Movement Therapist Tina Margareta Nilssen based on the clear understanding of the needs, challenges, and motivations of performing musicians. It complements traditional music tuition by educating musicians on efficient instrument-specific body mechanics, aims to improve performance-related movement control, and provide practical, music- and instrument centered sessions. It is positively framed by putting the emphasis on the enhancement of the playing mechanics, but also supports musicians with existing playing-related injuries. Detailed information is provided about the method itself in the “Materials and Methods” section.

The method is relatively new, therefore there is little data available. The Norwegian Academy of Music’s Centre for Excellence in Music Performance Education (CEMPE) produced an unpublished report (Breian and Jørgensen, 2015) based on a 3-month long intervention with six participants, concluding that the method helped with avoiding performance-related injuries, and the participants found it highly relevant to their own instrumental practice. There are two additional Master theses (Danielsen, 2013; Skorstad, 2015) and a Bachelor thesis (Brænden, 2015) written on the topic with similar results, but a more thorough and deeper examination of the method is deemed necessary.

The present study is the first large-scale study exploring the impact of Timani on individual musicians. The method can serve both as an intervention for injuries and as an educational, preventative strategy, similarly to other methods reported in the literature, where there is no clear differentiation made between treatment and preventions (Stanhope et al., 2020). Therefore, we did not selectively choose participants with or without performance-related problems but decided to include young musicians from the Norwegian Music Academy’s (NMH) talent program (TUP), and BA and MA classes, who are preparing for their professional careers for two reasons. Firstly, the method aims to establish healthy body mechanics for practice and performance, which is more effective in younger musicians because they have fewer established habits (Cruder et al., 2020). The importance of delivering health-related interventions in this generation is stressed in the literature (Spahn et al., 2014; Rickert et al., 2015) to shape their future practice behaviors which can have a significant impact on their careers. Secondly, research shows that there is a high percentage of performance-related musculoskeletal disorders (PRMDs) and other performance-related problems already in college-level music students and the problems are most frequent in the 1st and 2nd year Masters students (Cruder et al., 2020). This means that the selected participants are highly vulnerable to obtaining PRMDs or other performance-related health problems in the close future, so can hugely benefit from getting support during their professional education in establishing healthy habits.

We aimed to explore the impact of the intervention on performance-related problems, performance quality, and since the method provides practical solutions to support the playing technique, we also hypothesized that it would influence performance-related self-efficacy. Furthermore, we also wanted to list and assess the pedagogical tools used to achieve the outcomes.

Originally, the intervention was planned to run in-person in Oslo, but due to the Norwegian Government’s COVID19 regulations, the study had to be moved online. This change was challenging but also provided an opportunity to gather information about how this somatic method can be taught in a virtual environment.

Following the events of 2020 and 2021, the research exploring online teaching exploded as it became the primary way of education during the pandemic, and researchers explored several different angles and settings (Saikat et al., 2021). However, to the best of our knowledge, no studies are yet available on how somatic methods can be taught online, which seems extremely sensitive in terms of the need for personal contact. Somatic methods often use physical touch to guide the student or client, and the teachers also need to be able to observe the movements closely and from different angles. In the absence of these basic conditions, teaching somatic methods online can be challenging.

Under the given circumstances, therefore, an additional research question was added to the study, namely, examining the effectiveness of online education in the case of Timani.

Our research questions were the following:

(1) How can Timani support instrumental technique, the body mechanics when playing an instrument and the self-efficacy of the musician?
(2) Can Timani be effective in reducing performance-related problems?
(3) What tools and strategies are used to contribute to the outcomes?
(4) Can Timani be effectively taught online?

The study is the result of a collaboration between the Musician’s Health and Movement Institute (Oslo) and the University of York (United Kingdom).

Funding was generously provided by the White Rose College of Arts and Humanities as part of the Ph.D. funding of the first author.

## Materials and Methods

### Procedure

This is the first large-scale research study examining the effects of Timani on individual musicians. The exploratory nature of the study prompted a mixed methodology approach with strong qualitative elements. The main data collection was performed during a 4-week long intervention program and an opening and closing workshop. The participants filled out a questionnaire at the beginning of the first workshop and at the end of the closing workshop. The questionnaire collected basic demographic data and focused on two main areas: performance-related pain, injury or difficulties, and performance-related self-efficacy. Two validated scales were used to measure these constructs, the Musculoskeletal Pain Intensity and Interference Questionnaire (Berque et al., 2014) and the Music Performance Self-Efficacy Scale (Zelenak, 2014). To gather more information about the participants’ overall experience, self-constructed questions were added to the closing questionnaire, focusing on the participants’ engagement with the material, perceived outcome, and practice behaviors.

The intervention itself provided the participants with four, 45 min long individual Timani sessions online (one session each week) from a certified Timani teacher, which were observed by the researcher. Out of the 68 administered lessons, the researcher observed 63 (some sessions ran parallel) with her camera and microphone turned off to avoid any interference with the teaching process. The targets of these observations were the following: perceived effectiveness of the Timani exercises (i.e., changes in the posture and instrumental technique of the student), pedagogical tools, communication, feedback from the student, and use of the online environment. After each session, all participants were asked to fill out a short form with two open, qualitative questions regarding their experiences with the lesson, and their experiences with the online environment, which resulted in 68 short entries. To add an additional viewpoint, the teachers who administered the lessons were also interviewed after the conclusion of the project about their pedagogical approaches, the perceived impact and effectiveness of their teaching, and the use of the online space.

The research was conducted in two languages: Norwegian and English. The choice of language in different situations was informed by the level of understanding of the participants. The teachers and the researcher are bilingual, which allowed the lessons to be conducted in the participant’s first language in each case to maximize clarity, and they were encouraged to provide the written qualitative feedback in the same language. The interviews with the teachers were conducted in English and the observational notes were taken both in English and Norwegian. The translations of the qualitative data were done by the researcher.

### Participants

The participants for this study were recruited from the Norwegian Academy of Music (NMH) and its talent program for young musicians (TUP). The initial call for participants was made through the Academy, but the interested participants and their guardians were asked to contact the Musician’s Movement and Health Institute directly. Nineteen students were recruited, but due to scheduling difficulties and a drop-out, the study was completed with 17 students [mean age: 19.17 years, 9 females, 8 males; 9 attending the talent program, and 8 students from the Norwegian Academy of Music’s (NMH) Bachelor and Masters programs in performance]. More information about the participants is shown in Table 1.

**TABLE 1:** Participant demographics.

	Instrument	Gender	Age	Program
(1)	Violin	F	14	TUP
(2)	Violin	F	14	TUP
(3)	Cello	M	12	TUP
(4)	Cello	F	13	TUP
(5)	Saxophone	M	18	TUP
(6)	Saxophone	M	19	TUP
(7)	Trombone	M	16	TUP
(8)	Trombone	M	17	TUP
(9)	Organ	M	15	TUP
(10)	Cello	F	22	NMH
(11)	Flute	F	21	NMH
(12)	Flute	F	22	NMH
(13)	Clarinet	F	20	NMH
(14)	Harp	F	30	NMH
(15)	Piano (jazz)	F	24	NMH
(16)	Organ	M	24	NMH
(17)	Conducting	M	25	NMH

### Ethical Considerations

The participants were all young musicians, seven of them minors. Ethical approval was obtained from the Arts and Humanities Ethics Committee at the University of York, and the Norwegian Research Council was notified of the project in alignment with the regulations. In order to protect the personal data of the participants, the researcher did not obtain the full names or email addresses of the participants or, in the case of minors, the email addresses or full names of the parents or caregivers. All communication, including sending out the information sheets, collecting the signed consent forms, and scheduling the lessons was done by the four teachers assigned to teach the students. Moreover, all students entered the Zoom calls only using their first names. This way, the researcher did not obtain or store any personal information and was not in direct contact with the participants or the guardians of the participants. However, the participants and guardians were encouraged to communicate with the teacher in case any question or concern arose, so these could be forwarded to the researcher anonymously.

### Timani – The Method

Timani is a recently established somatic method for musicians that has been developed since 2007 by the Norwegian pianist, Massage Therapist, yoga teacher, Kinetic Control Movement Therapist and personal trainer Tina Margareta Nilssen.

Timani provides an analytical tool to recognize playing-related, compensatory movement patterns, and an approach to change less effective habits into more sustainable and functional movement patterns, teaching anatomy and using targeted, practical exercises. For the most part, the exercises are initially carried out away from the instrument, and then immediately integrated into the playing or singing with awareness of both the body and the produced sound.

The knowledge about anatomy and the more than 100 exercises are designed to help musicians understand and overcome challenges related to pain, injury, technical issues, sound production, or even just to enhance one’s playing to access higher potential as a musician. By focusing on the physiological aspects of playing or singing, it is aimed to enable musicians to make the most effective use of their bodies while practicing and performing. The method is used by musicians worldwide – from soloists to entire ensembles, and from students to established professionals – whether they have already experienced performance-related pain or injury or want to prevent them arising in the first place. Timani is also becoming increasingly popular in schools and colleges, taught by an ever-increasing network of teachers in 15 countries.

In a standard Timani session the students get: (1) a simple analysis of their playing or singing, and an anatomical explanation of relevant muscles related to the analysis, (2) targeted exercises (normally 1 – 3 exercises per session) to access and coordinate muscles or increase sensory stimuli (enhancing proprioception) that can expand one’s movement options while playing, and (3) implementation of the new awareness or activation while playing or singing. The exercises normally don’t require any equipment but are performed with conscious awareness of a body part, muscle, or movement in order to activate and become aware of new movement options that are made available for using while playing or singing.

The foundation of Timani is based on seven pillars:

(1) Relevant anatomy for musicians – Knowing basic anatomy and biology that provides an understanding of the complexity of muscular action and movement needed for playing or singing. One learns about key muscles that are important for musicians and exercises to differentiate and coordinate these muscles for the purpose of performing music. For example, learning about and becoming aware of the function of the intrinsic muscles of the hand can create more clarity of how to balance these muscles for more effective movements. Or learning which muscles to relax and which muscles to use for controlling shoulder movement can offload some of the muscles that usually tends to compensate for a lack of stability in the area.
(2) Movement analysis for musicians – Learning how to observe certain movement patterns and be able to determine if the coordination is sustainable or straining for your body.
(3) The fascia system – Fascia is an important tissue in the body that connects us from top to toe. To gain an effortless technique, some basic understanding of fascia is needed, as it provides elasticity and connection in our movements.
(4) Healthy natural breathing – Accessing healthy breathing is dependent on the functioning of the whole body. Learning which muscles are involved in breathing and how to access better lung capacity and breath control is essential for winds and singers.
(5) Getting to know ground force reaction – Some of the exercises are aimed at creating a new relationship to contact with the floor (feet), the chair (sit bones), and the instrument (fingers, hands, and arms). These are based on developmental movements and the natural way that we trained our movements in early developmental stages.
(6) Brain-body connection – The brain plays an essential role in the movement as movement happens as a communication between the sensory and motor system, each of which participates equally in developing accurate movement skills.
(7) Awareness – Awareness can make us more connected to the musical intention and the physical experience of playing. What we are not aware of, we cannot control or change. Awareness and focus are essential to creating changes in already automatic movement patterns.

### The Teachers

The four teachers in this study were all certified and experienced Timani teachers who received their education in the method Timani from the Musician’s Health and Movement Institute. They had a dual role, firstly, they acted as teachers throughout the study, administering the online lessons, secondly, they took up the role of participants after the study was concluded and were interviewed by the researcher. The semi-structured interview schedule used for these interviews were constructed based on the observations and were directed at the pedagogical approaches used, the perceived success of the teaching, and the adaptation to the online environment. They received an information sheet and signed a consent form before the interviews were scheduled.

### Expertise of the Researcher

The researcher is a professional flutist with an MA performance degree. In preparation for this study, she completed 6 months of the first year of the Timani teacher qualification at the Musician’s Movement and Health Institute prior to the data collection and observed Timani classes to gain a better understanding of the method itself.

### Analysis

**FIGURE 1:**
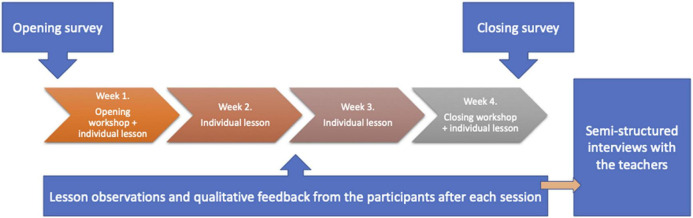
The process of the data collection.

Following Creswell’s (2003) criteria for mixed-method research designs, four main steps were taken into consideration during designing the study and analyzing the data: implementation, priority, integration, and theoretical perspective. Implementation refers to the sequence in which the methods are used in the overall design, priority addresses the question of which source of data is chosen to be superior in case there are opposing concepts emerging, integration is the process of connecting data from different sources, and the theoretical perspective governs whether the theory informs the analysis from the beginning, or it is emerging during the research process. In our research design, the survey at the beginning and the end of the intervention served as a frame for most of the qualitative data collection: the observations and the feedback from the students. The last part of the data collection, the semi-structured interviews with the teachers, were informed by the observations and the feedback as presented in Figure 1. Priority was given to the qualitative data because not all concepts targeted by the inquiry are measurable reliably in a quantitative way, such as perceived performance quality.

Integration is one of the most important processes in triangulation in mixed methodology research (Fielding, 2012). In the process of integrating different types of data, two main approaches were taken. Firstly, the data obtained from the surveys, the observations, and the feedback were integrated after the conclusion of the data collection. Two topics for the qualitative analysis were signposted by the survey, performance-related self-efficacy and performance-related pain intensity and interference, therefore, qualitative data which seemed to be linked to these concepts were highlighted and collected into a separate category whenever possible to allow for the triangulation. Secondly, the qualitative data from the observations and the feedback were used to inform the semi-structured interview schedule, thus, we obtained more information about the already emerged categories, while allowing the interviewees to introduce new topics.

In our theoretical approach, we had our target topics to examine, therefore the theory, namely that the Timani intervention will have an impact on the participants’ instrumental playing, self-efficacy, and performance-related pain, explicitly informed the analysis. However, there were some new and unexpected topics emerging, which were included in the final analysis.

## Results

The findings of this study came from the triangulation of the four different sources: the lesson observations, the qualitative feedback from the students, the main questionnaire administered at the beginning and at the end of the research, and the interviews with the teachers.

In the following section, the findings from each of these sources will be presented, and afterward, used to create a coherent model. Each of these sources highlights the intervention from a different angle, providing information on the student’s personal experience, the teachers’ experience, an outsider view, and a measurable, quantitatively shown change. The data answering the third research question about the online environment will be presented separately, under a different subheading.

### Survey Data

Two validated questionnaires were used in the pre-and post-intervention survey, measuring performance-related self-efficacy and performance-related injury.

The Music Performance Self-Efficacy Scale (Zelenak, 2014) has four subscales, mastery experiences, vicarious experiences, verbal/social persuasion, and psychological state. While the repeated measures ANOVA showed no significant differences between the pre-and post-measures in the overall score, there was a significant difference in the subscale ‘vicarious experiences’ before the intervention (*M* = 59.587, *SD* = 27.902) and after the intervention (*M* = 77.267, *SD* = 18.339) (*t* = 2.337, *p* < 0.05).

The Musculoskeletal Pain Intensity and Interference Questionnaire (Berque et al., 2014) collects data about lifetime, 12 months, 1 month, and past week prevalence, and participants who experienced performance-related pain in the past month are invited to fill out questions about the pain intensity and its interference with their mood and everyday life. Out of the 17, 12 participants reported experiencing performance-related pain in their lifetime, and 7 suffered from it in the month preceding the data collection. We analyzed the data coming from this subset and found that there was a significant difference between the measure before the intervention (*M* = 3.387, *SD* = 1.580) and after the intervention [(*M* = 1.397, *SD* = 1.639); *t* = 4.216, *p* = 0.006].

We added self-constructed questions to the final survey, inquiring about the participants’ overall experience with the intervention, their practice behavior during the 4 weeks, if they intended to use the gained knowledge in their private practice in the future, and how well it complements their instrumental tuition.

All 17 participants felt that the intervention had a positive impact on them; the qualitative entries about the overall experience were focused on professional outcomes, but also reported physical and psychological changes. In some form, all participants noted that they achieved better performance results with less effort, increased endurance, and enhanced sound production.

*I have found more stability and a smoother and bigger sound. And the pianissimo became more transparent as well and it does not sound so low.*

*I feel that I’m finding my own tone more and more, and I can actually get the sound that I want without wearing myself out.*

Many noted that they felt more confident when performing, experienced less anxiety, and were able to focus on their performance goals and musical expression more.

*I felt inner calmness when I played. Also, less nerves.*

The questions directed at the practice behavior during the intervention showed that four participants practiced the subscribed Timani exercises every day, 10 participants every other day, and 3 participants twice a week. All 17 participants had the intention to continue with the exercises, some even sharing plans to seek out further lessons. When asked about how they can incorporate the new information into the lessons with their primary instrumental teachers, the participants shared that they gained a new insight into how to solve specific performance problems they have already been working on in their lessons. Moreover, they felt that with the help they received during the intervention, they will be able to meet their teachers’ expectations better.

*We work very often to get a freer sound, and we always work to have enough air, but I feel that we never take the body as a starting point with my instrumental teacher. We work according to what we hear and try different things, but often there is a lot of focus on visualizing a story or character you want to play out. I have actually learned a whole new lesson about how breathing works in the sessions and it helped me a lot.*

*I think that the exercises give me a better technique and stability, which gives me the surplus to perform the musical things my teacher asks for.*

There were differences in the participant’s plans for using the exercises in the future: some said that they will return to them from time to time, while others were planning to use them frequently.

*I will definitely use this in the future. Timani provides an insight into how the body works when we are playing, which in turn gives more back from the hours you put into practicing.*

### Qualitative Feedback

Each participant was invited to answer two open questions immediately after each session, one asking about their general experience with the lesson, and the other regarding their experience with being taught online. Thus, each participant submitted four pieces of feedback, resulting in 68 short entries, the lengths spanning from a single sentence to full paragraphs. Most participants described the exercises done on the lesson, following with their experience with them and the resulting changes. Some of the entries touched on more than one topic, while others focused on only one specific experienced outcome. Twelve entries were only descriptive or too general (e.g., “*The Timani lesson was very good today”*), therefore we excluded them from the analysis.

After conducting qualitative analysis, six categories were established: (1) grounded, (2) easy to play, (3) improved sound production, (4) psychological benefits, (5) physical benefits, and (6) embodied knowledge.

The first category included 33 comments about feeling more balanced and supported, getting better contact with the chair or the floor, and positive bodily sensations. Twenty-four comments regarding the second category highlighted how the playing itself became less laborious, more natural, and required less effort in general. The third category included 19 pieces of data talking about improvements in the sound production and instrumental technique, such as more stable and even sound, which was easier to control. Some participants noted that the changes following the exercises were more noticeable in the musical output than in bodily sensations. The fourth category links closely to the second research question about performance-related pain: participants were reporting reduced tension and reduced sensations of pain while playing in 17 entries. The fifth category, labeled “psychological benefits,” collected 12 comments about feeling calmer and more self-confident but also feeling seen and understood. The final group of themes included all 11 comments about the embodied learning experience; some participants stated that it was “cool to learn things about the body,” and they appreciated that they received practical knowledge which supported their playing. Some representative quotes for each category can be found in Table 2.

**TABLE 2:** Qualitative feedback categories.

	Category and frequency	Quotes
(1)	Grounded (33 comments)	“After the exercise, I felt more grounded, and stopped fidgeting around.”
(2)	Easy to play (24 comments)	“It is much easier to play now, and I even get a better sound.”
(3)	Improved sound production (19 comments)	“I feel like I got access to a steady and big sound in my playing without pushing and using a lot of extra force.”
(4)	Physical benefits (17 comments)	“The usual tension in my arms was gone, and my fingers felt very relaxed.”
(5)	Psychological benefits (12 comments)	“I loved the class today, it made me so calm and connected to the music!”
(6)	Embodied knowledge (11 comments)	“It is so cool to learn these things, I feel my entire body much better.”

Two additional overarching meta-categories were formed, which both included the original six groups of themes. These deal with the timeline of the experiences, with some reporting “revelations,” i.e., immediate changes in the experience, while others gradual development.

### Observations

During the intervention, the researcher took notes about every lesson focusing on the content, the communication, the feedback from the participant, the pedagogical tools, the perceived outcome, and the use of the online environment, producing over 120 pages of documentation. These were organized and analyzed in two different ways: firstly, the learning process of each participant across the four lessons, and secondly, week by week, looking at the overall progress of all participants. While the lessons’ contents varied greatly depending on the instrument and the participants’ needs, there were some typical characteristics. The teachers choose more postural exercises (both with and without the instrument) in the first lessons and tended to move toward addressing finer motor movements in the later ones. However, they always linked the content with the participants’ articulated wishes and technical issues and provided them with practical solutions.

The observational notes identified similar categories to the qualitative feedback: physical and professional benefits (i.e., changing in playing posture and enhanced sound production) were frequently perceived. The participants seemed to play more freely and naturally with fewer mistakes, and the breathing mechanisms of the wind players notably improved: they played through longer phrases without any visible effort.

These outcomes were achieved by various tools, which were sorted into four categories: first and foremost, the educational content, which was based on the dual expertise in music performance and performance-informed body mechanics with a strong focus on the musical outcomes as well as biomechanical ones. Secondly, the constructivist and flexible structure of the sessions that alternated between instrumental playing and physical exercises, thirdly, the communication, which was student-centered and age-appropriate, and lastly, the pedagogical tools used, namely, demonstrations and visual aids.

The structure of the sessions was characterized by alternation between playing and physical exercises, which allowed the participants to test the impact of each exercise on the instrument and observe any changes which took place. This process was supported by student-centered and open communication: teachers asked for the student’s feedback after each segment of the session. A large proportion of these questions directed the students’ attention toward their own bodily sensations after each exercise and each short playing session, stressing that there is no right or wrong answer. This eliminated any desire to please the teacher by giving the “right” response, helped their concentration and cultivated self-exploration and curiosity toward their own body mechanics, and enhanced the students’ autonomy and agency. It also provided the teacher with clear feedback, and they could make the judgment whether the exercise was appropriate for the student. Moreover, the teachers avoided abstract language or metaphors as much as possible and used visual aids, such as skeleton models, anatomical pictures, and short videos to clarify their points. Demonstrations of the exercises were also frequent in the sessions to support the oral description of the activity.

### Interviews

The four semi-structured interviews were conducted immediately after the conclusion of the intervention study. The interview schedule included questions about the teachers’ backgrounds, the pedagogical tools used, including lesson planning, the communication with the students, especially in relation to their age, the perceived outcome, and their experience with the online environment. A thematic qualitative analysis was performed on the transcribed interview texts, which produced the following themes: personal experience, positive impact, communication, and planning.

All teachers highlighted their own personal experience with the method, stating that it proved to be a concise, relevant, and positive approach for enhancing their own professional playing experience, and articulated the need to support other musicians with their newly found knowledge:

*I started realizing for myself how much of a difference it made. And … you just can’t keep it to yourself.*

The listed positive impacts of the intervention appeared to happen on different levels, firstly, they observed immediate changes in postural alignment, body mechanics, and playing quality. They were, however, much more hesitant to make claims about the long-term impact of the intervention, stating that it is highly dependent on the participants’ personality and circumstances.

*Sometimes, you can do an exercise once and they go like: wow! And they go home, and they do that every day, and it sort of just clicks, and with other students, you can have this ‘aha’, and then they have forgotten.*

The interviews, however, identified another type of long-term impact which, to some of them, seemed even more important, namely, making the knowledge available:

*Now it is natural that this knowledge exists, they have tried it […] and if they have any problems they will know what to do. Because then they have this sense that there are answers, there are people who know. So, if I need something, there is somewhere that I can go. And that’s even more important to me than just solving a shoulder problem if you know what I mean.*

Following the theme “structure” identified by the observations, the teachers were asked about planning the sessions. They all seemed to take a constructivist and flexible approach with little pre-determined material. They prepared supporting material, such as anatomical pictures, the exercise manual available to all Timani teachers, and some general ideas based on the previous sessions, but most of the teaching content was informed by the feedback and playing of the participants.

*I have found it more difficult for me to teach, and I don’t often get good results with the student if I think that I know how it should go in the lesson. Because it’s kind of like… expectations on me, some unspoken expectations on them, and I prefer to sort of just take it as it comes.*

When asked about the communication, they all reported having no difficulty with interacting with the participants, and that they could get their ideas across. They all felt that this specific age group, especially the minors, sometimes required a slightly different vocabulary to make it more age-appropriate but this did not change the content of the sessions. However, they noted that the way they communicated was impacted more by the personality of the participant than their age.

*I feel that it is more person-dependent rather than age-dependent.*

### Overall Model

**FIGURE 2:**
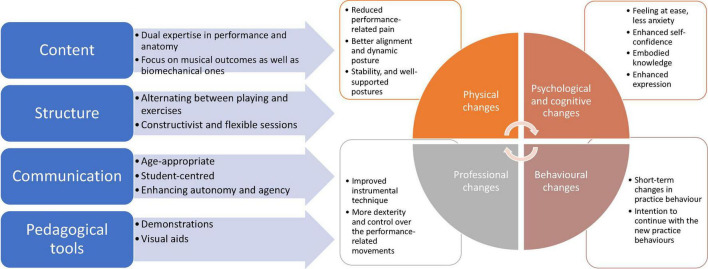
Overall model.

The integration of all the data resulted in an overarching model, which has two main parts: the impact of the intervention and the tools used to achieve it. The model is presented in Figure 2.

The impact of the intervention resulted in physical, professional and, to a certain extent, psychological, and behavioral changes. The performance-related pain and discomfort reduction as a result of the intervention was shown quantitatively but also was supported by the participant’s feedback. The observational data, the feedback and the teachers’ interviews all supported the hypothesis that Timani can improve the dynamic posture when playing.

Professional and technical benefits were shown by all qualitative data including enhanced sound production, more efficient playing technique, and more control and dexterity in the performance-related movements after the intervention.

There were some comments about psychological changes from the participants both during the lessons and in the written feedback about feeling calmer and experiencing less anxiety after the intervention. Also, many noted in the qualitative feedback that as the result of the intervention, they feel more self-confident that they can reach their performance goals. These comments were linked in the data to the quantitatively measured self-efficacy, which showed a significant change in the subscale “vicarious experiences.”

Due to the length of the intervention, we can only report some behavioral changes which took place during the intervention and cannot provide longitudinal data. The teachers’ interviews reinforced that whether someone includes the Timani exercises into their instrumental playing practice is highly dependent on the participants’ personality and circumstances. However, the qualitative data from the closing survey shows that all the participants have the intention to engage with Timani exercises in their daily practice.

### Online Environment

Teaching the method online had both advantages and disadvantages, and some of these were highly dependent on the individual’s environment and opportunities. Probably most important of all, in online learning, both the teacher and the student are dependent on the right infrastructure, including a strong internet connection and functional devices. Apart from a few occasions, these basic requirements were met. In the qualitative feedback, many participants mentioned that being able to study from the comfort of one’s home is a positive aspect. However, the observational data showed that this might be a disadvantage in cases where the student lacked privacy and was distracted by family members moving or talking in the background.

The other challenge, which was both observed and commented on by the participants, stemmed from the visuals the teachers are getting on the student. Ideally, the teacher should be able to observe the student from different angles. In a traditional, personal setting, this is easily solved: the teacher can change position while the student is playing. In this online environment, the student had to be asked to change the camera position and repeat the excerpt to capture the ideal angle, which, in some cases, disturbed the flow of the lesson. It seems that these difficulties did not result in the decreased quality of the lesson delivery, in fact, one of the participants noted that they were positively surprised how easy it was to follow the guidance from the teacher. The same positive feedback was repeated in the qualitative forms:

*Honestly, I’m very impressed by how well the teacher spotted small details through the screen.*

While most Timani exercises can be done without equipment or external physical guidance, there are a few exceptions and some basic needs to be met. One of the most frequent problems was the quality of the chairs available in the students’ homes and the practice rooms they signed in from, and the available space needed for the exercise.

The online environment also had some advantages such as the lack of traveling which made the scheduling easier and being able to learn from the comfort of one’s home. Also, the teachers were able to use various teaching materials tailored to the content of the lesson on the spot. As an example, they were sharing screens to show anatomical pictures or short videos to enhance their teaching, which was very well received by the participants.

Based on the observation and the qualitative questions, we concluded that the online format was an efficient method for teaching Timani.

## Discussion

The analysis and integration of the different types of data support the idea that the somatic method Timani can benefit instrumental technique, posture, and body mechanics. We found limited evidence to support our hypothesis that it can enhance performance-related self-efficacy, but the data suggest that it can decrease playing-related pain and discomfort. Moreover, the online delivery was satisfactory, and even created additional advantages for both the students and the teachers.

The final model summarizing the overall findings shows the positive impact on four different levels: physical, professional, and to a certain extent, psychological, and behavioral. These different layers deeply influence each other, and in some cases, overlap, and some suggestions of these effects are going to be made in the following paragraphs.

On a physical level, the findings show a positive change between pre-and post-intervention in terms of performance-related pain, posture, and body mechanics. The word ‘posture’ in this context refers to dynamic skeletal alignment both in an inactive and performance situation (Krasnow et al., 2001), while ‘body mechanics’ or ‘biomechanics’ is used to describe the active interaction with the instrument, i.e., movement patterns and coordination skills associated with the performance.

The students who had performance-related pain at the beginning of the study reported a decrease in their discomfort, which – in the qualitative data - they linked to the biomechanical and postural changes prompted by the exercises. The close relationship between posture, body mechanics, and performance-related problems is well established in the literature (Steinmetz et al., 2010). A biomechanically correct posture allows the loads to evenly distribute between the skeleton, musculature and joints which decreases the possibility of overloading and overuse (Chan and Ackermann, 2014), and it can also reduce performance-related fatigue (Hafez, 2019), therefore is an important factor in preventing playing-related physical injuries (Chan and Ackermann, 2014).

The importance of postural alignment and correct body mechanics as a means to treat and prevent performance-related problems cannot be understated, however, these tools can have a significant impact on healthy musicians as well: participants without pain also reported feeling more grounded and more at ease. Many linked these positive embodied feelings to psychological changes as well, such as ‘feeling calmer and more focused’ and reduced anxiety. Enhancing embodiment through somatic methods can result in reduced levels of performance anxiety; the phenomenon has been shown in several interventions using the Alexander technique (Hoberg, 2008; Klein et al., 2014).

More generally, modulating the body’s position can have a meaningful impact on mood regulation (Veenstra et al., 2017) and plays a part in reducing anticipatory anxiety (Lipnicki and Byrne, 2008; Weineck et al., 2020) and mental health in general (Domingues, 2018), moreover, it can improve interoception (Weineck et al., 2020). While many studies use gross postural differences to examine the phenomenon, such as slumped or erect and standing or supine conditions, it is quite possible that smaller changes in the posture might lead to similar benefits, which, given the high levels of performance anxiety and other mental health concerns in this population, might be a valuable tool.

Both physical and psychological effects were often observed and/or reported in connection with the performance quality, given that in Timani, the new movement coordination, muscle activation, and posture is immediately translated to the instrument. The corrected posture or body mechanics led to various performance outcomes, such as more dexterity in the upper extremities, better sound production and breathing mechanism, resulting in superior performance. This observation is also supported by the literature: the importance of body mechanics in instrumental playing was proposed as early as 1952: Polnauer (1952) argues that fully understanding the biomechanical process of the sound production would not only help avoid injuries, but also shorten training time, and improve musical skill and quality. His ideas were repeated more recently as well, linking posture to sound and performance quality (Ueno et al., 1998; Kreutz et al., 2008; Shoebridge et al., 2017).

More specifically, there is evidence of the relationship between breathing mechanisms and postural alignment: Ackermann et al. (2014) showed that variations in posture change the expansion of the chest and abdomen when playing a wind instrument, therefore, it is quite possible that which misalignments in the body can lead to reduced breathing capacity and disrupted air control (Chan and Ackermann, 2014). In Baadjou et al.’s (2017) study, clarinetists reported better sound quality and ‘feeling of more space or capacity to breathe’ (p. 106) after a postural correction. The reported and observed dexterity and more controlled and coordinated fine motor movements in the fingers and the arms can also be a result of the enhanced body mechanics: a more supported trunk leads to decreased muscle activation in the upper extremity, relieving unnecessary tension (Baadjou et al., 2017).

These positive performance outcomes prompted another notable change. In the qualitative data, many participants commented on becoming more self-confident in reaching their performance goals. This was prompted by gaining access to previously unknown, practical, and replicable tools that had an impact on their playing. Since self-efficacy is defined as a belief that the individual is able to perform the required task and reach the desired outcome (Bandura et al., 1977; Ritchie and Williamon, 2010), during the data integration process, we linked these qualitative entries about self-confidence to the results of the Performance-Related Self-Efficacy scale. It is quite interesting that while the qualitative entries frequently mention the topic when measured quantitatively, we were only able to show a significant difference in the PRSE in the subscale ‘vicarious experiences.’ The term refers to the process when the individual is enhancing their self-efficacy by observing others completing a task and modeling it (Hendricks, 2015). There might be several reasons why the participants showed improvement in this specific sub-scale, but the frequently used demonstrations in the sessions can be viewed as a contributor.

In conclusion, it seems that the intervention prompted changes in posture and body mechanics which supported the participants on three levels, physical, psychological, and professional, further signposting the importance of the inclusion of body mechanics in music education and professional practices.

Yet, resources such as postural analysis and instrument-specific and performance-informed body mechanical corrections are generally not readily available for musicians, even though there is a clear interest expressed toward the topic: in Ioannou and Altenmüller’s (2015) 68.7% of the student participants expressed that they believed that basic knowledge in anatomy and physiology would be a necessary part of music education, and 43.4% expressed that this knowledge would help them in avoiding performance-related problems.

In sports performance, the analysis of occupational biomechanics is a frequently used technique to enhance performance and has given rise to valuable insights over the years (McGinnis, 1999; van der Kruk et al., 2018; Taborri et al., 2020). In music, in spite of the success in incorporating the tool into music education and performance in a handful of studies (Kjelland, 2000; Visentin et al., 2008; Kelleher et al., 2013), the primary source of information about the instrumental or singing technique, including the posture and the specific movement patterns needed for the sound production, is the instrumental or singing teacher, whose primary focus is the auditory output, rather the supporting body mechanics (Chan and Ackermann, 2014). However, all musical tasks, such as the musical expression or specific techniques, are underpinned by a unique muscular activation and in the absence of specific anatomical knowledge, teachers often use their own technique or their education as a model (Mills and Smith, 2003; Visentin et al., 2008). Also, their access to new potential approaches and ideas can be restricted due to the relative isolation they work in, and the prevailing model of master-apprentice tuition (Haddon, 2009). While in some cases this can be an effective model for music tuition, it does not necessarily account for anthropometric differences. As an example, differences in the length of the neck can have implications for the headrest used in violinists, or the facial structure might inform the shape of the ideal mouthpiece. Therefore, replicating the teacher’s specific technique might not always be ideal for the student.

Moreover, in instrumental or singing lessons, metaphors are often used to communicate and describe the desired movement coordination, and these can be interpreted in many ways. As an example, instructions such as ‘play with a heavy arm’ can provide useful insights for some students, but others might over-activate muscles in the bowing arm to achieve the effect, which can result in inferior sound production and control. With this in mind, it seems necessary for musicians to access anatomically informed support regarding their posture and body mechanics when playing to avoid injuries and enhance their instrumental or singing technique. The challenge is to translate efficient biomechanics into a dynamic playing situation, which needs combined expertise in performance and anatomy (Shoebridge et al., 2017).

The characteristics of the Timani sessions in our research demonstrated how these two can be blended and administered in an efficient and personal manner. The observational data and the interviews with the teachers provided an insight into the tools used to achieve the noted positive physical and professional outcomes.

The structure of the sessions, namely, alternating between exercises and playing created an atmosphere similar to an instrumental teaching session which the participants seemed to be very comfortable with. Research shows that music students favor practical, instrument-specific and individual injury prevention programs (Stanhope, 2018), and are more likely to be engaged with the material when they find it relevant and directly related to their instrumental playing (Ackermann et al., 2002), and the sessions seemed to satisfy this need.

Also, excessive focus on postural corrections divorced from the playing can be a barrier to achieving positive results: in Shoebridge et al.’s (2017) study musicians felt that such postural interventions separated them from the expression, and they were “losing sight of performance as the goal of coordination” (p. 832). In the observed Timani sessions, the relevance of the exercises to the playing was made clear by observing the quality of the sound and the ease of expression supported by the new movement behavior as well as the change in the instrumental technique and the overall physical sensations in the body. In a sense, all the physical work done in the sessions was informed by the performance goals. As a result, many participants reported that they were able to focus on the music more once their movement patterns and postural alignment was optimized.

This positive framing, placing the physical activity in the context of enhancing performance skills seemed to inspire and motivate the participants greatly. When the narrative is framed negatively, i.e., avoiding injury, the strongest predictor for engaging in some kind of physical activity seems to be experiencing symptoms or witnessing another musician suffering from an injury (Stanhope, 2018).

Opposing this, in our study, the participants seemed intrinsically motivated and enthusiastic, highlighted the professional benefits most frequently in their feedback, and diligently engaged with the exercises, including them in their practice routine. This behavioral benefit was consistent across the sample in the course of the intervention, but to examine whether this behavioral change is permanent, a longitudinal study would be necessary. According to the teachers’ interviews, the long-term outcome varies depending on the client’s personality, physical needs, and playing situation, but it seems promising that the participants in this study all had the intention to continue learning and practicing Timani exercises.

The oscillation between playing and physical exercises might have additional benefits. In exercise programs where the content of the intervention is divorced from the playing-related movement behavior, the positive effect might not translate well to practice and performance situations, especially the exertion levels (Chan et al., 2014a). Moreover, when discussing dynamic-playing related postures, Shoebridge et al. (2017) highlighted the importance of “rebalancing the self with the instrument and performance environment” (p. 821).

When probing further into the structure and the content of the sessions, the teachers described the procedure of leading the session and choosing appropriate material as a flexible and constructivist process. From an epistemological viewpoint, constructivism means that the knowledge is formed through the learner’s active interaction with the educational material, rather than a process of acquiring knowledge that exists outside the learner as an abstract entity (Shively, 2015). A constructivist approach also implies student-centeredness by actively involving the students in their own learning process, which was achieved by verbal and non-verbal communication, characterized by repeated open questions and adjusting the taught material based on the participant’s feedback in the sessions. Allowing the participant’s experience to guide the session steps away from the traditional master-apprentice model, and provides autonomy and agency for the student. Moreover, it seems to cultivate curiosity and engagement with the material.

In conclusion, this preliminary study suggests that Timani may have a significant impact on musicians’ performance-related body mechanics, and as a result, on the sound production, and additionally, might influence psychological states and practice-related behaviors. Its most unique characteristics, namely, that the postural alignment and motor coordination are taught in the context of instrumental playing seems to motivate musicians to engage with the physical exercises. Moreover, since it is created and taught by performing musicians or therapists with a special interest in working with musicians, it has a clear understanding of the challenges and difficulties of playing or singing on an advanced level, and the shortcomings of the generally provided educational frameworks. It aims to fill this gap with anatomical knowledge, but without losing sight of the performance goals. As the study demonstrated, it can have a very beneficial effect on young musicians, including the reduction of performance-related pain, the enhancement of instrumental technique, and providing accessible tools to reach one’s musical potential.

### Limitations

This has been an exploratory study with the aim of capturing multiple angles of the intervention. Therefore, some of these measures could have been more in-depth to provide more detailed information. We were also relying strongly on the participant’s self-report, which carries the risk of bias: young students might want to please the teachers and be less outspoken about their experiences. Nevertheless, the observational data supported the qualitative feedback from the students.

Also, performance is a complex phenomenon, and it is hard to measure its quality. The individual’s perception of their own playing can be influenced by several factors, such as the environment or their relationship to the teacher. The chosen method of self-report and observations might over-or underestimate the changes in quality and cannot be fully objective. In the future, to obtain more objective, quantitative measures, the scope of the inquiry will probably need to be narrowed down to a certain instrument type and/or specific techniques.

## Conclusion

The somatic method Timani brings a new approach to the field of musicians’ health and wellbeing. Among its many unique characteristics, the ability to transfer the impact of the physical exercises directly to the instrument seems to enhance motivation by experiencing immediate results in the playing technique itself. Musicians can easily merge these techniques directly into instrumental practice since the equipment needs are low, and the knowledge provided is practical and relatable.

While the importance of preventing PRMDs and other performance-related problems in this vulnerable population is indisputable, the method also highlights the role of body mechanics and posture in performance quality and offers a new avenue to enhance playing technique. This positive framing seems to increase the motivation to engage with the method among young musicians.

More research is needed to explore the long-term impact of the method and test its effectiveness in different ages and professional levels.

## Data Availability Statement

The raw data supporting the conclusions of this article will be made available by the authors, without undue reservation, to any qualified researcher.

## Ethics Statement

The studies involving human participants were reviewed and approved by the Arts and Humanities Ethics Committee of the University of York. Written informed consent to participate in this study was provided by the participants, and in the case of minors, by the participants’ legal guardian/next of kin.

## Author Contributions

AD responsible for the research design, the data collection and analysis, and the writing of the majority of the manuscript. TN organized the intervention, recruited the participants and the teachers, collected consent forms, and contributed to the manuscript with the description of the method and other corrections. Both authors contributed to the article and approved the submitted version.

## Conflict of Interest

The authors declare that the research was conducted in the absence of any commercial or financial relationships that could be construed as a potential conflict of interest.

## Publisher’s Note

All claims expressed in this article are solely those of the authors and do not necessarily represent those of their affiliated organizations, or those of the publisher, the editors and the reviewers. Any product that may be evaluated in this article, or claim that may be made by its manufacturer, is not guaranteed or endorsed by the publisher.
